# SensEURCity: A multi-city air quality dataset collected for 2020/2021 using open low-cost sensor systems

**DOI:** 10.1038/s41597-023-02135-w

**Published:** 2023-05-26

**Authors:** Martine Van Poppel, Philipp Schneider, Jan Peters, Sinan Yatkin, Michel Gerboles, Christina Matheeussen, Alena Bartonova, Silvije Davila, Marco Signorini, Matthias Vogt, Franck René Dauge, Jøran Solnes Skaar, Rolf Haugen

**Affiliations:** 1 Flemish Institute for Technological Research (VITO), Boeretang 200, 2400 Mol, Belgium; 2 The Climate and Environmental Research Institute NILU, PO Box 100, 2027 Kjeller, Norway; 3 European Commission, Joint Research Centre (JRC), Via Enrico Fermi 2749, 21027 Ispra, VA Italy; 4 Flanders Environment Agency, Dokter De Moorstraat 24-26, 9300 Aalst, Belgium; 5 Institute for Medical Research and Occupational Health, Ksaverska cesta 2, Zagreb, Croatia; 6 Liberaintentio Srl, Malnate, 21046 Italy

**Keywords:** Environmental monitoring, Scientific data, Design, synthesis and processing, Imaging and sensing

## Abstract

Low-cost air quality sensor systems can be deployed at high density, making them a significant candidate of complementary tools for improved air quality assessment. However, they still suffer from poor or unknown data quality. In this paper, we report on a unique dataset including the raw sensor data of quality-controlled sensor networks along with co-located reference data sets. Sensor data are collected using the AirSensEUR sensor system, including sensors to monitor NO, NO_2_, O_3_, CO, PM_2.5_, PM_10_, PM_1_, CO_2_ and meteorological parameters. In total, 85 sensor systems were deployed throughout a year in three European cities (Antwerp, Oslo and Zagreb), resulting in a dataset comprising different meteorological and ambient conditions. The main data collection included two co-location campaigns in different seasons at an Air Quality Monitoring Station (AQMS) in each city and a deployment at various locations in each city (also including locations at other AQMSs). The dataset consists of data files with sensor and reference data, and metadata files with description of locations, deployment dates and description of sensors and reference instruments.

## Background & Summary

Air quality remains a major concern in many parts of Europe, especially in urban areas^1^. The most important air pollutants in terms of health are particulate matter (PM), nitrogen dioxide (NO_2_), and ground-level ozone (O_3_).

For the efficient implementation of air policies, air quality monitoring data with high spatial density and temporal resolution, and with sufficient quality are needed; These data can supplement data from Air Quality Monitoring Stations (AQMSs) that are used to assess the ambient air quality in Europe as defined in the Directive 2008/50/EC.

Low-cost air quality sensor systems consist of an integrated set of hardware that uses one or more sensors to measure the quantity of a chemical species and can supply real time measurements^2^.

Thanks to their lower cost than the reference air quality monitoring methods^3^ sensor systems can be deployed at high density, making them a significant candidate of complementary tools for improved air quality management. However, they still suffer from poor or unknown data quality^3^. Sensor signals can be affected by interfering compounds, temperature, humidity, pressure and signal drift over time^4–6^.

**Fig. 1:**
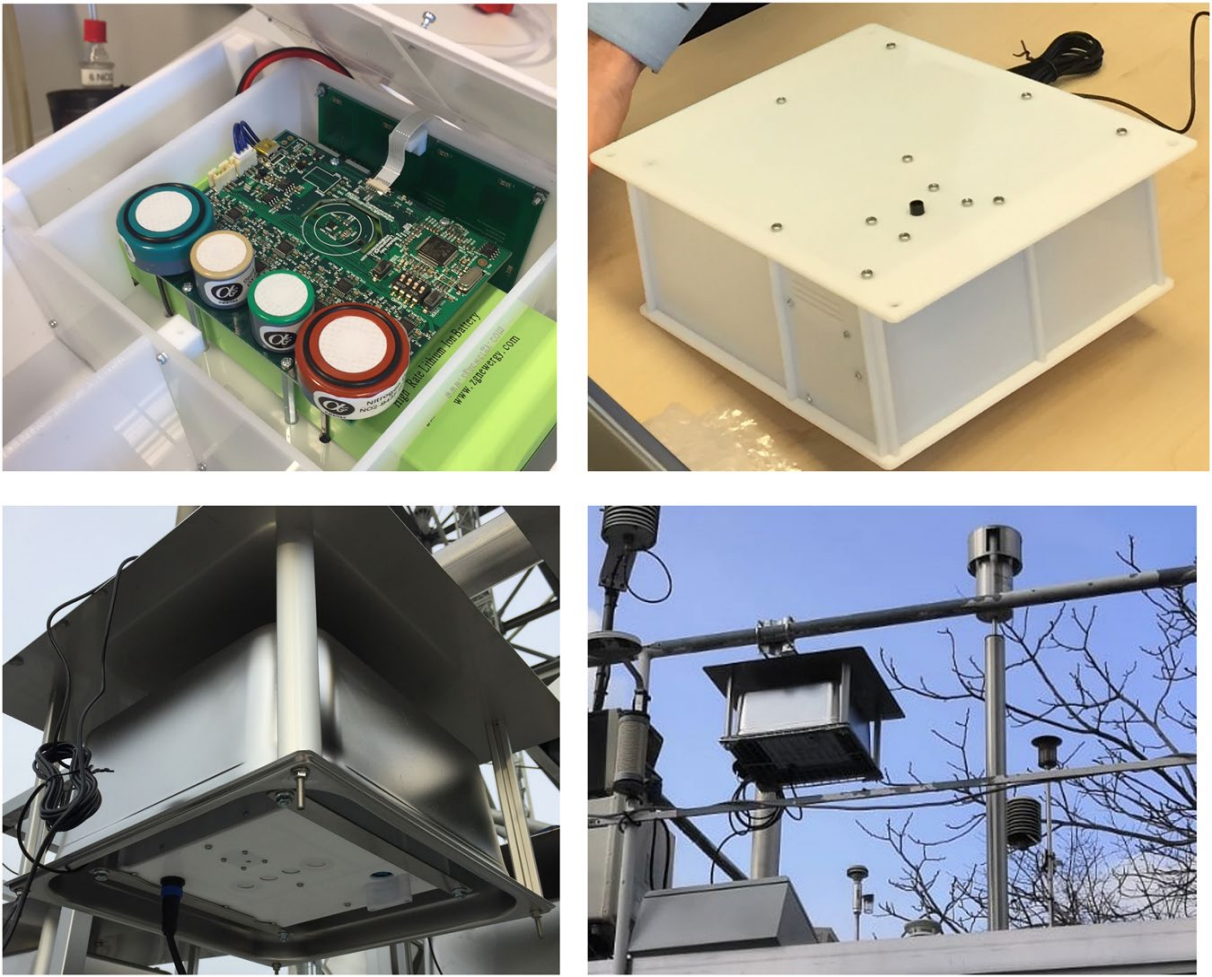
AirSensEUR sensor systems: (top left) chemical shield with gas sensors; (top right) sensor box with sampling inlet of OPC-N3 on top; (bottom left) sensor box in outdoor enclosure with gas sensors and PMS5003 sensor inlet and (bottom right) sensor box installed at Air Quality Monitoring Stations.

The European Commission - Joint Research Centre (JRC) has recently conducted a research project to evaluate low-cost sensor system, namely the AirSensEUR, as a supplemental tool for reference air quality monitoring. The AirSensEUR sensor system (Fig. 1) contains sensors to monitor NO, NO_2_, O_3_, CO, PM_2.5_, PM_10_, PM_1_, CO_2_ and meteorological parameters (temperature, relative humidity, and atmospheric pressure). Within this project, the air quality data from the 85 AirSensEURs and partially from the Air Quality Monitoring Stations (AQMSs) were collected. The 85 sensor systems were deployed throughout a year in three European cities, namely Antwerp (Belgium)-34 systems, Oslo (Norway)-34 systems, and Zagreb (Croatia)-17 systems. The aims of this project are to obtain insight in the performance evaluation of low-cost sensors under different meteorological and ambient conditions, and to explore and evaluate calibration approaches^7^.

In this paper, we report a unique dataset including the raw sensor data of quality-controlled sensor networks along with comprehensive reference data sets when the sensors are co-located at an AQMS. With permission of the European Commission, we make these data available for the research community to enable further research without the need for the very time- and resource-consuming process of collecting the data themselves.

Although it is possible to find many articles^8^, reports (https://vaquums.eu/deliverables) in literature or web pages(https://web.jrc.ec.europa.eu/rapps/pub/aqsensors/, http://www.aqmd.gov/aq-spec/evaluations#&MainContent_C001_Col00=2, https://www.epa.gov/air-sensor-toolbox) about the accuracy of sensor systems, to the best of our knowledge there is no substantial open dataset published, presenting collocated raw sensor data and reference measurements except one study reporting data from twelve particulate matter sensors collocated with beta-attenuation PM mass monitor for three months^9^. Research topics may include but are not limited to developing and testing calibration models for air quality sensors, implementing correction algorithms, and evaluating the sensor performance under different environmental conditions. The data may help to develop and evaluate drift of calibration models and to a better understanding of sensor performance triggering advancement in sensor technology which may result in improved sensor data quality.

The main data collection period lasted from April 2020 until April 2021, but not all sensor systems acquired data for the entire period. The sensor systems were first co-located at a reference AQMS in each city, then deployed at various locations in the city and after that co-located again at the same reference AQMS.

**Fig. 2:**
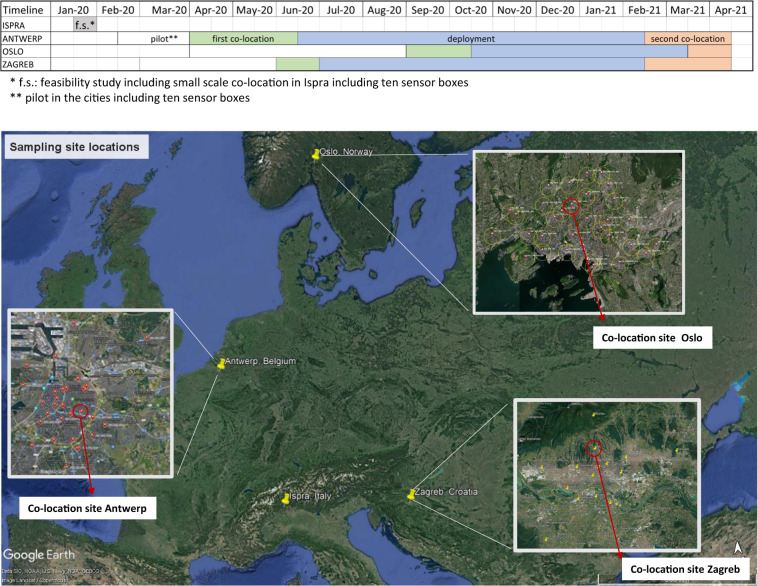
Overview of the sampling with sampling site locations and timeline. Map data ©2022 Google Imagery ©2022 Nasa, Terrametrics.

An overview of the timeline and sampling sites is given in Fig. 2.

Broad ranges of meteorological conditions and pollutant concentrations were covered during co-locations and deployment.

The deployment sites in each city show a good spatial distribution within the city and are characterised by different impact of traffic. Some of the sites are the AQMSs and were selected to observe correspondence with reference data over a longer time. In addition, some sites were selected very close to each other to assess short-term spatial variability. At some of the AQMSs, duplicate sensors were deployed to evaluate the between sensor variance.

## Methods

### AirsensEUR sensor systems

The sensor systems used in this study were AirSensEUR version 3.0^7^. AirSensEUR is an Open Platform project developed by the JRC in collaboration with Liberaintentio S.r.l (IT), aimed at measuring air quality accurately using low-cost sensors (LCSs). Both hardware schematics and the software running on the units, or the calibration procedures implemented as a post-processing of the collected data are described under public licenses (https://github.com/ec-jrc/airsenseur-sensorsshield, https://github.com/ec-jrc/airsenseur-sensorshost, https://github.com/ec-jrc/airsenseur-box, https://github.com/ec-jrc/airsenseur-box).

The data collected from the units were stored locally and periodically sent to external server to an InfluxDB database^7^ for offline post-processing and/or calibration. The data were generally transferred via GPRS/LTE and via WiFi connections for a few units.

AirSensEUR includes a PTFE enclosure with a size of 26 cm × 22 cm × 10 cm and a weight of 2 kg, battery included (see Fig. 1 top). The PTFE enclosure was inserted in a stainless-steel protecting cover. The overall size of protective stainless-steel cover is 35 cm × 32 cm × 30 cm except for the top cover, which is made from a 42 cm × 45 cm aluminium plate (see Fig. 1 bottom).

Table 1 gives an overview of the measured pollutants, the sensor type and manufacturer. The OPC-N3 has 24 size bins (0.3/0.35–40 µm) with a counting efficiency of 50% @ 0.3 μm and 100% @ 0.35 μm and the PMS5003 has 6 size bins (>0.3 µm) with a counting efficiency of 50% @ 0.3 µm and 100% @ 0.5 µm. Both counting efficiencies were claimed by the manufacturers although it was shown by experiments that the counting efficiency for the PMS5003 sensor is about 80% at 0.5 µm^10^. No publications on counting efficiencies of OPC-N3 are available to our knowledge.

**Table 1:** Sensors included in the AirSensEUR sensor systems including sensor variables as provided in the dataset.

Parameters	Supplier- Sensors	Type	Raw data column header	Raw units
Atmospheric pressure	Bosch Sensortec - BMP280	Piezo-resistive	BMP280	hPa
Ambient Temperature	Sensirion - SHT31	Semi-Conductor	SHT31TE	°C
Ambient Relative humidity	Sensirion - SHT31	Semi-Conductor	SHT31HE	%
Internal Temperature	Sensirion - SHT31	Semi-Conductor	SHT31TI	°C
Internal Relative humidity	Sensirion - SHT31)	Semi-Conductor	SHT31HI	%
CO	Alphasense - CO-A4	Electrochemical	CO_A4_P1	nA
CO_2_	ELT - D-300-3	NDIR	D300	ppm
NO	Alphasense - NO-B4	Electrochemical	NO_B4_P1	nA
NO_2_	Alphasense - NO2-B43	Electrochemical	NO2_B43F_P1	nA
O_3_	Alphasense - OX-A43	Electrochemical	OX_A431_P1	nA
PM_10_	Alphasense - OPC-N3	Optical Particle Counter	OPCN3PM10	µg/m^3^
PM_2.5_	Alphasense - OPC-N3	Optical Particle Counter	OPCN3PM25	µg/m^3^
PM_1_	Alphasense - OPC-N3	Optical Particle Counter	OPCN3PM1	µg/m^3^
Number of particles	Alphasense - OPC-N3	Optical Particle Counter	OPCN3Bin0, OPCN3Bin1, OPCN3Bin2,…, OPCN3Bin23	counts/mL
PM_10_	Plantower - PMS5003	Nephelometer	5310CAT, 5310CST	µg/m^3^
PM_2.5_	Plantower - PMS5003	Nephelometer	5325CAT, 5325CST	µg/m^3^
PM_1_	Plantower - PMS5003	Nephelometer	5301CAT, 5301CST	µg/m^3^
Number of particles	Plantower - PMS5003	Nephelometer	53PT003, 53PT005, 53PT010, 53PT025, 53PT050, 53PT100	counts/0.1 L

The gas sensors for NO_2_, CO, NO and O_3_ are installed on the AirSensEUR Chemical sensor Shield (version R31), PM and CO_2_ sensors are installed on the Exp1Shield R10 sensor shield^7^. In addition, the sensor box is equipped with sensors for monitoring temperature and relative humidity inside the AirSensEUR box nearby chemical sensors and other sensors for monitoring ambient air temperature, relative humidity and atmospheric pressure outside the AirSensEUR box on a Flyboard. The information presented in Table 1 is also given in the metadata file metadata_sensors.csv^11^.

### Sensor box sampling periods

The data were collected between April 2020 and April 2021. The exact sampling intervals in each city were slightly different. An overview of the timeline of the sampling is given in Fig. 2. The Figure shows the dates of the feasibility study in Ispra, the pilot studies in the cities, the first co-location in the cities, the deployment at different sites in the cities and second co-location in the cities. Details on the sampling is given in the paragraph below. A detailed overview of the start and stop dates at each location is given in metadata file (File metadata_dates.csv^11^).

Prior to the sampling campaigns in the cities, selected sensor systems were deployed at the EMEP-ABSIS-ICOS station of the JRC in Ispra (IT) as an initial feasibility study. Subsequently, the pilot studies were performed prior to the sampling campaigns in each city with the same ten boxes of the feasibility study, to test data transfer, installation, etc. In each city, the sampling campaigns included three consecutive phases:

- co-location of all sensor systems at an AQMS in the city (hereafter called ‘first co-location’)
- deployment of the sensor systems at different locations of the same city (hereafter called ‘deployment’)
- co-location of all sensor systems at the same AQMS of the first collocation (hereafter called ‘second co-location’)

#### Feasibility study in Ispra and pilot studies in the three cities (Antwerp, Oslo and Zagreb) prior to the main co-locations and deployment campaigns

Ten sensor systems were installed at the EMEP-ABSIS-ICOS station of the JRC in Ispra (IT), a semi-rural site in Northern Italy^12^, between 17 and 31 January 2020 (Fig. 2). The same ten systems were used in the pilot study in the three cities: four of the sensor systems were deployed in Antwerp (40641B, 4065D0, 4065E0 and 4065E3) and in Oslo (40458D, 40642E, 4065ED and 40816F) and two in Zagreb (4047D0 and 406414). The purpose of this study was to check the reliability of AirSensEUR sensor systems as well as to collect data for calibration at a semi-rural site. The characteristics of the reference air pollution analysers and meteorological parameters at the EMEP-ABSIS-ICOS station are given in Table 2. The gas analysers were routinely calibrated, and daily calibration checks were performed to detect and correct possible drifts of the monitoring equipment.

**Table 2:** The reference analysers used at the AQMSs.

Parameters	Technique used	Type	Units
ISPRA			
PM_10_	Oscillating Microbalance	Thermo Environment TEOM 1405 FDMS	µg/m^3^
NO	Chemiluminescence	Thermo Environment 42i	ppb
NO_2_	Cavity attenuated phase shift spectroscopy (CAPS)	Aerodyne caps NO2	ppb
CO	Non-dispersive Infrared Gas-Filter Correlation Spectroscopy	Horiba APMA - 370	ppm
O_3_	Ultraviolet photometry	Thermo Environment 49i	ppb
CO_2,_ sampling at 30 meter high	Wavelength-Scanned Cavity Ring Down Spectroscopy (WS-CRDS)	Picarro G2401	ppm
ANTWERP ANT_REF_R801			
PM_10_	Optical particle counter	Palas Fidas 200	µg/m^3^
PM_2.5_	Optical particle counter	Palas Fidas 200	µg/m^3^
PM_1_	Optical particle counter	Palas Fidas 200	µg/m^3^
NO/NO_2_	Chemiluminescence	Thermo Environment 42i	ppb
CO	Non-dispersive IR spectroscopy	Teledyne API T300	ppm
O_3_	Ultraviolet photometry	Teledyne API T400	ppb
CO_2_	Non-dispersive IR absorption spectroscopy	Sick Sidor	ppm
OSLO OSL_REF_KVN			
PM_10_	Oscillating Microbalance	Thermo TEOM 1405 FDMS^(1)^	µg/m^3^
PM_2.5_	Oscillating Microbalance (equivalent method)	Thermo TEOM 1405 FDMS^(1)^	µg/m^3^
PM_10_	Light scattering (equivalent method)	Palas Fidas 200^(1)^	µg/m^3^
PM_2.5_	Light scattering (equivalent method)	Palas Fidas 200^(1)^	µg/m^3^
PM_1_	Light scattering (equivalent method)	Palas Fidas 200^(1)^	µg/m^3^
NO/NO_2_	Chemiluminescence (reference method)	Opsis Serinus 40	µg/m^3^
CO	NDIR spectroscopy (reference method)	Opsis Serinus 30	ppm
O_3_	UV photometry (reference method)	Teledyne API T400^(1)^	ppb
ZAGREB ZAG_REF_IMI			
PM_10_	Gravimetry	Sven Leckel SEQ. 47/50-CD	µg/m^3^
PM_2.5_	Gravimetry	Sven Leckel SEQ. 47/50-CD	µg/m^3^
NO/NO_2_	Chemiluminescence	Horiba APNA −370	µg/m^3^
CO	Non-dispersive IR spectroscopy	Horiba APMA - 370	µg/m^3^
O_3_	Non-dispersive ultraviolet absorption (NDUV)	Horiba APOA – 370	µg/m^3^

The sensors systems used in the feasibility study in Ispra were also included into initial pilot studies in Antwerp, Oslo and Zagreb before the first co-location in order to check the correct deployment and operation at a few field sites (see Fig. 2 and file metadata_dates.csv^11^).

#### Common naming-convention for sampling site description

A common naming-convention for sampling site description for the three consecutive phases in the three cities is used. The sampling site labels (IDs) are of the form is XXX_YYY_ZZZ(Z) with:

- The XXX referring to the city: ANT (Antwerp); OSL (Oslo); ZAG (Zagreb);
- The YYY describing the type of location: URB (Urban background or suburban background); TRA (Traffic site in urban or suburban area), RUR (Rural site), REF (AQMS with reference measurements, without any further characterisation);
- The three or four ZZZ(Z) referring to the street name of location, or the name of AQMS.

#### Main co-locations and deployment campaigns in antwerp

The sensor systems installation at the AQMS 42R801 of Borgerhout for the first co-location took place on 2020-04-02 and 2020-04-03, where the sampling lasted roughly until 2020-06-05 (about 72 days). Between 2020-06-15 and 2020-06-18, the sensor systems were moved to their deployment sites, apart from two units that were installed on 2020-06-22. The sensor systems stayed at the deployment sites for approximately 8 months. Between 2021-02-17 and 2021-02-26 the sensor systems were taken down from their deployment locations and installed at the same AQMS for the second co-location lasted until 2021-04-13 (lasted about 45 days). A detailed overview of the start- and stop dates at each location (deployment sites) is given in metadata file (File metadata_dates.csv^11^) and is visually displayed in Fig. 4.

#### Main co-locations and deployment campaigns in Oslo

The sensor systems involved in the first co-location exercise were installed at the Kirkeveien AQMS between 2020-08-26 and 2020-08-28, which the sampling lasted roughly until 2020-10-14 (about 48 days), except for two sensor systems (4065ED and 40458D) that stayed at the pilot sites (OSL_TRAF_VINK and OSL_TRAF_LIND). Subsequently, all units except for two, were moved to their deployment sites. The installation at the deployment sites started on 2020-10-16, and by 2020-12-01, most sensor systems were operational until 2021-03-08 (roughly 88 days). The installation of sensor systems for the second co-location took place on 2021-03-08 and 2021-03-10, which lasted until 2021-04-09 (roughly 31 days). One sensor system collected data over a very limited period. A detailed overview of the start- and stop dates at each location (deployment sites) is given in metadata file (File Metadata_dates.csv^11^) and is visually displayed in Fig. 6.

#### Main co-locations and deployment campaigns in Zagreb

The sensor systems installation for the first co-location at the IMI AQMS took place on 2020-05-18 and 2020-05-27, and co-location lasted roughly until 2020-07-15 (around 60 days). The deployment period was roughly between 2020-07-20 and 2021-02-18 (approximately 7 months). The second co-location lasted roughly between 2021-03-03 and 2021-04-12 (approximately 37 days). A detailed overview of the start- and stop dates at each location (deployment sites) is given in metadata file (File metadata_dates.csv^11^) and is visually displayed in Fig. 8.

### Co-location sites

The details of the AQMSs where the co-location campaigns took place including measured pollutants and reference analysers at each AQMS are given in Tables 2. In Oslo (OSL_REF_KVN), the Palas Fidas 200 data is reported only during the 1^st^ co-location and during the 2^nd^ co-location, the data from the TEOM instrument is reported instead of the Palas Fidas 200 data. For naming convention of the test sites in the Table 2, we refer to section “Sensor locations: deployment sites”.

#### Co-location site in antwerp

The AQMS of ANT_REF_R801 (Station 42R801- Borgerhout, see www.irceline.be, 4.43178°E, 51.20961°N, at an attitude of 10 m) is an urban background station installed at 30 m from the main road Plantin en Moretuslei.

The reference monitoring at ANT_REF_R801 includes PM_10_, PM_2.5_, NOx (NO_2_ and NO), CO, CO_2_ and O_3_, see Table 2. The reference station also includes SO_2_, BC and UFP monitoring. CO and CO_2_ monitoring are not permanently performed. During the two co-locations, one CO and one CO_2_ monitors were temporally installed at the station (same inlet as other gases). The list of AirSensEUR sensor systems co-located at the ANT_REF_R801 station are given in metadata_dates.csv^11^.

#### Co-location site in Oslo

The Kirkeveien AQMS (OSL_REF_KVN), located at 10.72455°E, and 59.93230°N at an altitude of 58.3 m, is a traffic station situated next to an urban ring road with an average daily traffic intensity of ca. 15,000 vehicles.

The reference monitoring at OSL_REF_KVN normally includes PM_10_, PM_2.5_, CO, NO and NO_2_. An O_3_ monitor was additionally installed at the station for two co-location campaigns. In addition to two TEOM PM monitors with PM_2.5_ and PM_10_ inlets, a Palas Fidas 200 instrument was also operational during the 1st co-location campaign. The list of AirSensEUR sensor systems co-located at the OSL_REF_KVN station are given in metadata_dates.csv^11^.

#### Co-location site in Zagreb

The IMI AQMS (ZAG_REF_IMI), located at 45.835305°N, 15.977822°E, at an altitude of 195 m, is an urban background station within the Zagreb network for air quality monitoring.

The reference monitoring at ZAG_REF_IMI includes PM_10_, PM_2.5_, NOx (NO_2_ and NO), CO, O_3_, SO_2_ and benzene. The list of AirSensEUR sensor systems co-located at the ZAG_REF_IMI station are given in metadata_dates.csv^11^.

### Sensor locations: deployment sites

**Fig. 3:**
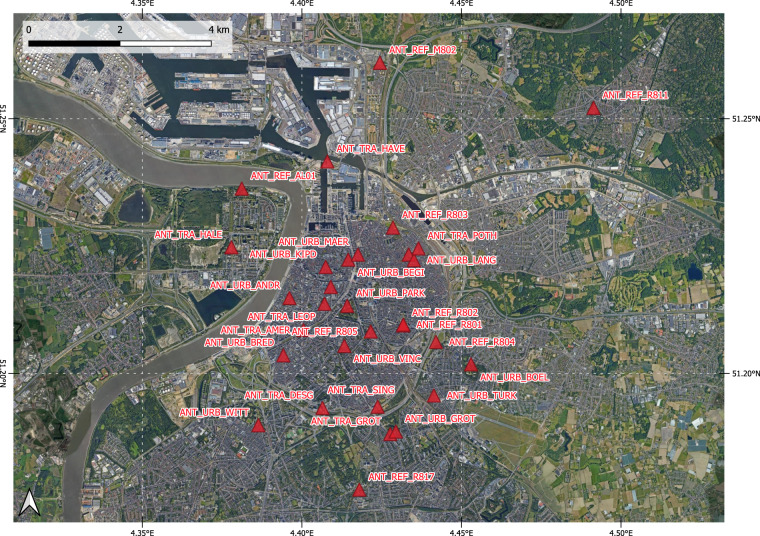
Sensor deployment sites in Antwerp. Imagery ©2022 Google, Imagery ©2022 TerraMetrics, Map data ©2022 Google.

**Fig. 4:**
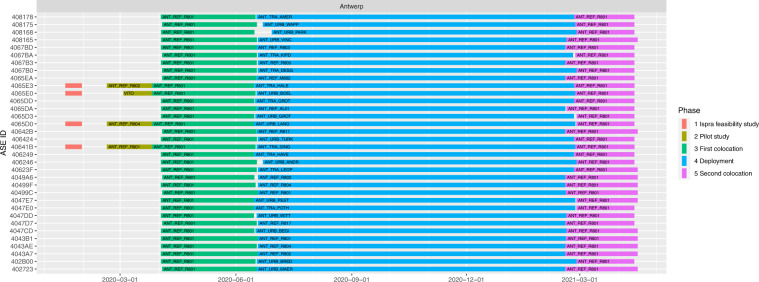
Timeline of sensor deployment in Antwerp, with sensor ID on left axis and location ID displayed in the colored bars that represent the different phases over time.

**Fig. 5:**
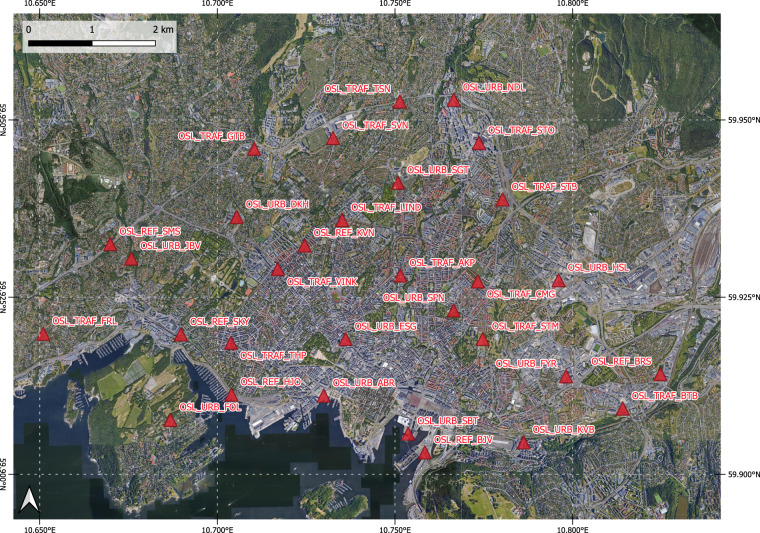
Sensor deployment sites in Oslo. Imagery ©2022 Google, Imagery ©2022 TerraMetrics, Map data ©2022 Google.

**Fig. 6:**
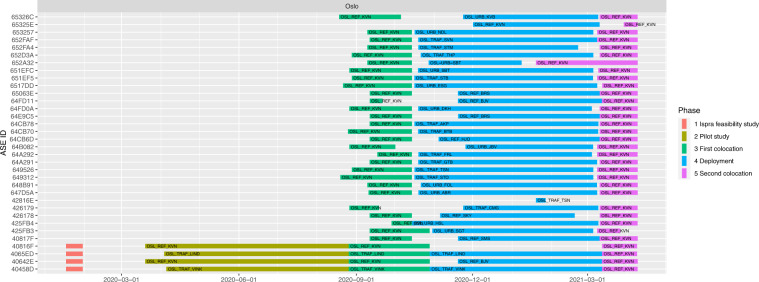
Timeline of sensor deployment in Oslo, with sensor ID on left axis and location ID displayed in the colored bars that represent the different phases over time.

**Fig. 7:**
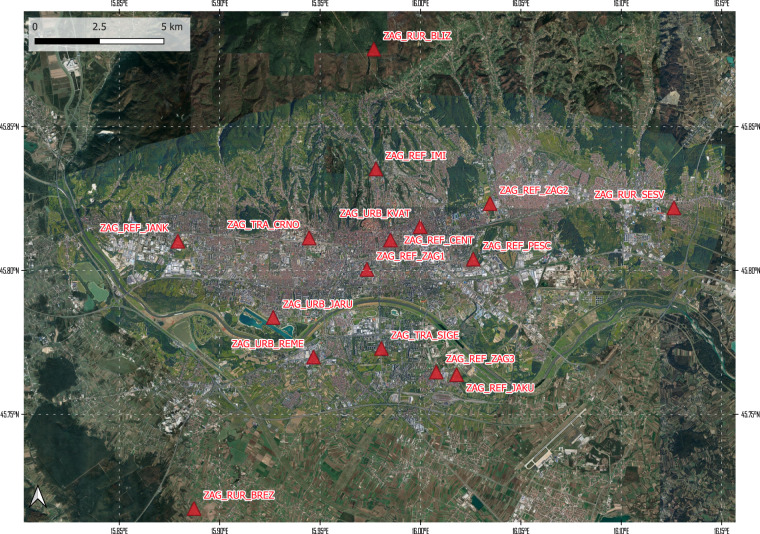
Sensor deployment sites in Zagreb. Imagery ©2022 Maxar Technologies, CNES/Airbus, Maxar Technologies, Google, Airbus, Imagery ©2022 TerraMetrics, Map data ©2022 Google.

**Fig. 8:**
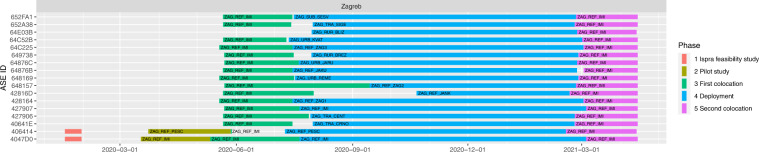
Timeline of sensor deployment in Zagreb, with sensor ID on left axis and location ID displayed in the colored bars that represent the different phases over time.

The sensor locations in the three cities for the deployment are given in Figs. 3, 5, 7. Detailed information of the timeline of the data collection for the different sensor systems and their locations is displayed in Figs. 4, 6, 8. The common naming convention of the form XXX_YYY_ZZZ(Z) is explained before under the heading ‘Common naming-convention for sampling site description’. The deployment sites were selected to assure a good spatial coverage over each city as well as a suitable distribution between background, traffic and AQMSs. The deployment sites are characterised by different impact of traffic: both in terms of traffic density as well as distance to the street. Most sampling sites are at other locations than an AQMS (further referred to as ‘dedicated sites’). Some sites were selected very close to each other (neighbouring sensors) with variation in traffic density to assess short-term spatial variability. Some sensor systems were installed at the AQMSs to check the agreement between sensors and reference analysers over a longer period than the co-location periods. At some of the AQMSs, the duplicate systems were deployed to evaluate the between sensor variances. In total, three, three and one duplicate sensor systems were installed respectively in Antwerp, Oslo and Zagreb. The duplicate sensor systems were respectively: 40499 C – 4043A7, 4049A6 – 4067BD, 4043AE – 4067B3 for Antwerp; 40642E – 64FD11, 64E9C5 – 65063E,649526 – 42816E for Oslo and 4047D0 - 427907 for Zagreb. The corresponding locations of these boxes during the deployment are given in metadata_dates.csv and the locations are described in metadata_sites.csv^11^. The number of sensor systems installed in each city and the distribution over AQMSs and ‘dedicated’ locations is given in Table 3.

**Table 3:** Total number of AirSensEUR sensor systems and their distribution at the AQMSs and dedicated sites during the deployment in the three cities.

	Antwerp	Oslo	Zagreb
AQ monitoring stations	9	6	8**
Sites with duplicate systems	3	3	1
Dedicated sites	22	24	8
Total number of systems	34	33	17

**In addition, two sensor systems were installed at the AQMSs which are not automatic stations but have filters or other sampling techniques (data of these stations are not used and therefore not classified as reference station).

The file metadata_sites.csv^11^ file contains the metadata of sampling sites in the three cities with the distances to road and an indication of traffic intensity. For Antwerp, the traffic intensity (vehicles per hour) was based on the modelled data of Department MOW (*Mobiliteit en Openbare werken* or Mobility and Public works), calculated from the annually averaged traffic density over all hours of the day, and therefore the actual vehicle numbers during peak hours may be much larger. For Oslo, the daily averaged traffic density was initially estimated using a traffic model and, then it was converted to averaged hourly traffic density to be consistent with the Antwerp data. For Zagreb, the quantitative traffic density information was not available, instead, the qualitative estimates were provided.

Detailed information of each sampling site is given as a pdf file (metadata_sampling_site_description.pdf^11^).

### Conditions during co-location and deployment

The ambient conditions and concentrations during the co-location campaigns and deployment showed broad ranges.

#### Meteorological conditions

A broad range of atmospheric conditions was covered during co-location and deployment in the three cities. An overview of the conditions is summarized in Table 4.

**Table 4:** Ranges of atmospheric conditions during the co-locations and deployment in the three cities ([min, max] hourly values for Oslo and Antwerp; daily values for Zagreb).

	Co-location		Deployment	
	T (°C)	RH (%)	T (°C)	RH (%)
Antwerp	[−1, 28]	[21, 99]	[−7, 39]	[20, 100]
Oslo	[−3, 26]	[25, 100]	[−14, 12]	[29, 100]
Zagreb	[0, 26]	[44, 89]	[−3, 27]	[33, 97]

During the co-location periods, the hourly temperature ranged between −1 and 28 °C in Antwerp and between −3 and 26 °C in Oslo. The daily averages in Zagreb were between 0 and 26 °C. The hourly relative humidity ranged between 25 and 100% in Oslo, between 21 and 99% in Antwerp and between 44 and 89% in Zagreb (daily values).

During the deployment, the hourly temperature ranged between −7 and 39 °C in Antwerp and between −14 and 12 °C in Oslo. The daily averages in Zagreb were between −3 and 27 °C. The hourly relative humidity ranged between 29 and 100% in Oslo, between 20 and 100% in Antwerp and between 33 and 97% in Zagreb (daily values).

The deployment in Oslo did not cover the summer period, resulting in a narrower range in atmospheric conditions.

#### Pollutant concentrations

The air pollutant concentrations measured at the AQMSs during the co-location campaigns showed broad ranges, with the maximum (hourly) concentrations of 175 μg/m^3^ O_3_ in Zagreb, of 114 μg/m^3^ NO in Oslo, of 139 μg/m^3^ NO_2_ in Antwerp and of 152 μg/m^3^ for PM_10_ in Antwerp.

During the deployment, the concentrations measured at the AQMSs showed also broad ranges, with the maximum (hourly) concentrations of 241 μg/m^3^ O_3_ in Zagreb, of 292 μg/m^3^ PM_2.5_ in Oslo and of 125 μg/m^3^ NO_2_and 312 μg/m^3^ NO in Antwerp.

Notable differences in concentrations were observed between the two co-locations and deployment periods as well as between the cities.

## Data Records

The data are publicly available and can be freely accessed from Zenedo^11^. The dataset consists of the data files (Directory dataset) and metadata (Directory metadata).

### Sensor and reference data

One data file is supplied for each AirSensEUR sensor system in csv format comma separated without quotes. The naming-convention of the data files is given as “City_ASE_ID.csv”, where City corresponds to the city where the AirSensEUR sensor systems were deployed, ASE stands for AirSensEUR and ID is a unique identifier of each sensor system. The data files are given in wide format with one row of data for each minute when the AirSensEUR sensor systems recorded any data of any sensors. Within each row, any missing data is reported with an empty field. Each row includes minute raw sensor data, reference data, meteorological data (temperature, relative humidity and atmospheric pressure), date, time and location. The column headers present in datasets are listed in Tables 5–7 with description and units for sensor and reference data. The datasets also include quality flags for sensor data as described in section Technical Validation.

**Table 5:** Description of date format, location and meteorological data present in all datasets of the AirSensEUR sensor systems.

*Header*	*Description*	*Format*
***date***	date time with ISO 8601^22^ format YYYY-MM-DDTHH:mm:ssZ. date corresponds to the POSIX current time of the Linux distribution running on the AirSensEUR sensor systems. The current time of Linux distribution is constantly updated through information retrieved by GPS and/or GPRS or WIFI. Transitory incorrect date may be observed until AirSensEUR sensor systems were synced with GPS/GPS or WIFI time. The time zone was set to Coordinated Universal Time for all the AirSensEUR used in this study	POSIX
***Latitude***	spherical latitude coordinate in decimal degree with up to 13 digits given by the GPS, SierraWireless GTOP Ladybird 1, included in AirSensEUR sensor systems	Decimal number
***Longitude***	spherical longitude coordinate in decimal degree with up to 13 digits given by the GPS included in AirSensEUR sensor systems.	Decimal number
***Altitude***	altitude in m with up to 13 digits given by the GPS included into the AirSensEUR sensor systems.	Decimal number
***LocationID***	when AirSensEUR box are co-located at any Air Quality Monitoring Station (AQMS), the LocationID identify the sampling with the Location ID given in Table 9 and metadata_sites.csv	string
***BMP280***	atmospheric pressure in hPa measured by the Bosch Sensortec BMP280 sensor. The sensor is located on the chemical sensor shield R31, see Fig. 1, top left	Decimal number
***SHT31HE, SHT31TE***	ambient relative humidity in % and temperature in °C, respectively, measured by the Sensirion SHT31-DIS-B sensor with filter membrane SF2. The sensor is located on a flying board directly sensing air with as little as possible influence from electronic heat and temperature of the stainless-steel protective box.	Decimal number
***Absolute_humidity***	Calculated quantity, the mass of water vapour in ambient air to the volume occupied by the air mixture in g/m³ that is, the concentration of water vapour that was computed using the Clausius–Clapeyron equation^23^.	Decimal number
***Td_deficit***	Calculated quantity, the difference between the ambient air temperature and the dew point in °C. The dew point was computed using the Magnus equation^24^.	Decimal bumber
***SHT31HI, SHT31TI***	relative humidity in % and Temperature in °C, respectively, measured by the Sensirion SHT31-DIS-B sensor. The sensor was located on the sensor shield R31, nearby the electrochemical sensors	Decimal number

**Table 6:** Description of air pollutant sensor data present in all datasets of the AirSensEUR sensor systems.

*Header*	*Description*	*Format*
***CO_B4_P1, NO_B4_P1, NO2_B431F_P1, OX_A431_P1***	raw sensor data for the CO-A4, NO-B4, NO2_B43F and OX-A431 sensors, respectively in nA measured at the sensor working electrodes	Decimal number
***D300***	raw sensor data for the D-300-3V sensor in ppm	Decimal number
***5310CAT, 5325CAT, 5301CAT***	PM_10_, PM_2.5_ and PM_1_ concentrations calibrated by the manufacturer in µg/m³ measured by the Plantower PMS 5003 sensor computed using the counts of the sensor bins and unknown algorithms of calibration.	Decimal number
***5310CST, 5325CST, 5301CST***	uncorrected PM_10_, PM_2.5_, PM_1_ concentrations in µg/m³ measured by the Plantower PMS 5003 sensor computed using the counts of the sensor bins and an unknown particles density	Decimal number
***53PT003, 53PT005, 53PT010, 53PT025, 53PT050, 53PT100***	number of particles with diameter over 0.3, 0.5, 1.0, 2.5, 5 and 10 µm, respective to the bin names in the headers, in 1 mL of air measured by Plantower PMS 5003 sensors	Decimal number
***OPCN3PM10, OPCN3PM25, OPCN3PM10***	PM_10_, PM_2.5_ and PM_1_ concentrations in µg/m³ measured by the OPC-N3 sensor computed using the counts of the sensor bins and a particles density of 1.65 kg/L	Decimal number
***OPCN3Bin0, OPCN3Bin1, OPCN3Bin2,…, OPCN3Bin23***	number of particles with diameter over 0.35, 0.46, 0.66, 1.0, 1.3, 1.7, 2.3, 3.0, 4.0, 5.2, 6.5, 8.0, 10, 12, 14, 16, 18, 20, 22, 25, 28, 31, 34 and 37 µm, respective to the bin names in the headers, per mL of air measured by an OPC-N3 sensor	Decimal number
***OPCN3Hum, OPCN3Temp***	relative humidity in % and temperature in °C measured in the PM chamber of OPC-N3 sensors	Decimal number
***OPCN3Vol***	volume of air in mL sampled by OPC-N3 sensors during each minute measurements	Decimal number

**Table 7:** Description of air pollutant reference data present in all datasets of the AirSensEUR sensor systems with their coordinates, temperature, relative humidity, and atmospheric pressure at their location.

*Header*	*Description*	*Format*
***Ref.Long***	spherical longitude coordinate in decimal degree with up to 7 digits for reference measurements.	Decimal number
***Ref.Lat***	spherical latitude coordinate in decimal degree with up to 7 digits for reference measurements.	Decimal number
***Ref.Press***	atmospheric pressure in hPa measured by the reference barometer of the reference station	Decimal number
***Ref.RH***	ambient air relative humidity in % measured by the reference sensor of the reference station	Decimal number
***Ref.Temp***	ambient air temperature in °C measured by the reference sensor of the reference station2	Decimal number
***Ref.CO_ppm***	reference CO measurements data, the reference analyser and unit are given in Table 2	Decimal number
***Ref.CO2***	reference CO_2_ measurements data, the reference analyser and unit are given in Table 2	Decimal number
***Ref.NO***	reference NO measurements data, the reference analyser and unit are given in Table 2	Decimal number
***Ref.NO2***	reference NO_2_ measurements data, the reference analyser and unit are given in Table 2	Decimal number
***Ref.O3***	reference CO measurements data, the reference analyser and unit are given in Table 2	Decimal number
***Ref.PM10***	reference PM_10_ measurements data, the reference analyser and unit are given in Table 2. Ref.PM10 is always included, provided that the data exists. However, additional columns may be included indicating the reference analyser used, e.g., Ref.PM10.TEOM, Ref.PM10.Fidas … according to the availability of these data.	Decimal number
***Ref.PM2.5***	reference PM_2.5_ measurements data, the reference analyser and unit are given in Table 2. Ref.PM2.5 is always included provided that the data exists However, additional columns may be included indicating the reference analyser used, e.g., Ref.PM2.5.Fidas, Ref.PM2.5.TEOM … according to the availability of these data.	Decimal number
***Ref.PM1***	reference PM_1_ measurements data, the reference analyser and unit are given in Table 2. Ref.PM1 is always included provided that the data exists. However, additional columns may be included indicating the reference analyser used, e.g., Ref.PM1.Fidas … according to the availability of these data	Decimal number

Note 1: in Oslo, all reference PM_10_ and PM_2.5_ mass concentrations given by FIDAS 200(S) and TEOM instruments were normalised using the slope and intercept of the regression line of daily TEOM and FIDAS data against daily data given by low volume samplers.

Note 2: In Antwerp during the 2^nd^ co-location, the reference temperature and reference relative humidity were given by the FIDAS 200 at site ANT_REF_R801 because the official meteo station was not operative anymore. It was checked that both temperature and relative humidity of the meteo station and FIDAS 200 correctly agreed during the 1^st^ colocation, with R² = 0.99, slope = 1 and intercept = 0.3 for temperature and R² = 0.94, slope = 0.95 and intercept = −1.2 for relative humidity.

In addition to the mass concentrations, particle numbers per bin of Palas Fidas 200 are supplied for the colocation site ANT_REF_801 during the 2 co-location periods and during deployment period in Antwerp (ANT_REF_R801_FIDAS_UTC.csv) and at OSL_REF_KVN in Oslo during the first co-location period (OSL_REF_KVN_Fidas_UTC.csv). The files are comma separated with minute data. The content of these files is described in Table 8. Missing data and invalid data are indicated with empty cell while 0 indicates no particle counts.

**Table 8:** Description of data present in ANT_REF_R801_FIDAS_UTC.csv and OSL_REF_KVN_Fidas_UTC.csv.

Header	Description	Format
***date***	POSIX date time with ISO 8601^22^ format YYYY-MM-DDTHH:mm:ssZ. date corresponds to the local time of the Fidas instrument transformed into Coordinated Universal Time	POSIX
***bin1…bin78***	Number of particles in Particles/cm³ in bin1 to bin 78. The diameters of particles associated with each bin is given in metadata_Fidas_um.csv.	Decimal number

### Metadata

Five metadata files are provided to describe:

- the sensor used in the AirSensEUR sensor systems (metadata_sensors.csv);
- the brand name of reference analysers (metadata_sites.csv) used at all sampling sites;
- additional data of the sampling sites, including e.g. location description, positioning of the sensor systems, picture of deployment (metadata_sampling_site.pdf);
- the sampling dates of the feasibility study, pilot studies, first and second co-location and deployment for all AirSensEUR sensor systems (metadata_dates.csv);
- and the diameters of particles associated with each bin (metadata_Fidas_um.csv).

The description of metadata in metadata_sensors.csv file is given in Table 1.

The description of metadata in metadata_sites.csv file is given in Table 9.

**Table 9:** Meta data for sampling sites.

Headers	Explanation	Format
location_id	Identifier of sampling site	String
city	City name	String
latitude_dd	Decimal latitude of sampling site in decimal degrees	Decimal number
longitude_dd	Decimal longitude of sampling site in decimal degrees	Decimalnumber
distance_to_road_m	Distance from the AirSensEUR box to the traffic lane in meter	Decimal number
average_hourly_traffic_intensity_number_per_h	Average hourly traffic intensity based on traffic models (for street next to sampling site)	Decimal number
notes	Comments	String
co_equipment, co2_equipment, no_Equipment, no2_equipment, o3_equipment, pm10_equipment, pm25_equipment, pm1_equipment	Identifier of routine reference analyser at AQMS for CO, CO_2_, NO, NO_2_, PM_10_, PM_2.5_ and PM_1_, respectively. All identifiers of reference analysers are consistent with the EIONET vocabulary given at http://dd.eionet.europa.eu/vocabulary/aq/measurementequipment/view?page=6#vocabularyConceptResults. There is one exception for the CO_2_ reference analyser at site ANT_REF_R801 in Antwerp labelled “SickSidor” (https://www.sick.com)	String
co_unit, co2_unit, no_unit, no2_unit, o3_unit, pm10_unit, pm25_unit, pm1_unit	Units for reference measurements for CO, CO_2_, NO, NO_2_, PM_10_, PM_2.5_ and PM_1_	String
other_pm10_equipment, other_pm25_equipment, other_pm1_equipment	At site OSL_REF_KVN where the 1st and 2^nd^ co-location took place, an additional PM analyser Palas Fidas 200 S was installed during the 1^st^ co-location and used for calibration of PM sensors	String
other_pm10_unit, other_pm25_unit, other_pm1_unit	Units for reference measurements for other_pm10_equipment, other_pm25_equipment, other_pm1_equipment	String

The description of metadata in metadata_dates.csv is given in Table 10.

**Table 10:** metadata for sampling periods.

Headers	Explanation	Format
ASEs	Identifier of the AirSensEUR sensor systems, six alphanumeric characters coded in base 16	String
City	City where the AirSensEUR system sensors are deployed (Antwerp, Oslo or Zagreb)	String
Ispra_Start	Date time with ISO 8601^22^ format *YYYY-MM-DD*T*HH:mm:ss*Z. It corresponds to the beginning of the feasibility study in Ispra for ten AirSensEUR sensor systems.	POSIX
Ispra_End	date time with ISO 8601^22^ format *YYYY-MM-DD*T*HH:mm:ss*Z. It corresponds to the end of the feasibility study in Ispra for ten AirSensEUR sensor systems.	POSIX
Pilot_Study_LocationID	Identifier of the sampling sites (see Table 9) where the pilot studies took place in the three cities for the ten AirSensEUR sensor systems involved in the feasibility study in Ispra. There is also a VITO site for the pilot tests	string
Pilot_Study _Start	Date time with ISO 8601^22^ format *YYYY-MM-DD*T*HH:mm:ss*Z. It corresponds to the beginning of the pilot studies.	POSIX
Pilot_Study _End	Date time with ISO 8601^22^ format *YYYY-MM-DD*T*HH:mm:ss*Z. It corresponds to the end of the pilot studies.	POSIX
First_Col_LocationID	Identifier of the sampling sites (see Table 9) where the 2020 1^st^ co-location took place for all AirSensEUR sensor systems in the three cities.	String
First_Col_Start	Date time with ISO 8601^22^ format *YYYY-MM-DD*T*HH:mm:ss*Z. It corresponds to the beginning of 1^st^ co-location in Three cities.	POSIX
First_Col_End	Date time with ISO 8601^22^ format *YYYY-MM-DD*T*HH:mm:ss*Z. It corresponds to the end of 1^st^ co-location in the three cities.	POSIX
Deployment_LocationID	Identifier of the sampling sites (see Table 9) where the main deployment took place for all AirSensEUR sensor systems in the three cities.	string
Deployment_Start	Date time with ISO 8601^22^ format *YYYY-MM-DD*T*HH:mm:ss*Z. It corresponds to the beginning of deployment in Three cities.	POSIX
Deployment_End	Date time with ISO 8601^22^ format *YYYY-MM-DD*T*HH:mm:ss*Z. It corresponds to the end of deployment in the three cities.	POSIX
Second_Col_LocationID	Identifier of the sampling sites (see Table 9) where the winter 2021 co-location took place for all AirSensEUR sensor systems in the three cities.	String
Second_Col_Start	Date time with ISO 8601^22^ format *YYYY-MM-DD*T*HH:mm:ss*Z. It corresponds to the beginning of 2^nd^ co-location in the three cities.	POSIX
Second_Col_End	Date time with ISO 8601^22^ format *YYYY-MM-DD*T*HH:mm:ss*Z. It corresponds to the end of 2^nd^ co-location in the three cities.	POSIX

## Technical Validation

### Quality assurance/control (QA/QC) procedures

During deployment sensor systems were regularly checked. In some cases, sensors had to be replaced or cleaned. For the reference data, common QA/QC procedures were applied consistent with the objectives of the European air quality directive (2008/50/EC) and conform with internationally accepted standards (EN ISO/IEC 17025); this means that the reference monitors at AQMS are serviced and calibrated on a regular basis and measurement uncertainties meet the Data Quality Objectives of the European air quality directive (2008/50/EC). The QA/QC described in relevant CEN standards (CEN 14211:2012 for NO/NO_2_^13^, CEN 14626:2012 for CO^14^, CEN 14625:2012 for O_3_^15^ and CEN 16450:2017 for PM_2.5_ and PM_10_^16^) were applied. In this paper, inconsistent sensor data were flagged. Data were flagged when certain threshold values are exceeded, indicating that the results are unreliable. In some cases, data were manually flagged based on knowledge from the field but without certain thresholds exceeded. The principle of data flagging is described below.

### Data collection and data flagging

Low-cost sensors may occasionally supply inconsistent data e. g. before reaching equilibria, when they are used out of the interval of temperature or humidity operation, under other extreme conditions, or simply when sensors are being transported. As such, a procedure including quality control and quality assurance (QA/QC) of the sensor data is necessary. In the following, a set of QA/QC and filtering steps is suggested, which has been used to provide quality flags in the datasets.

In all dataset files, columns giving sensor data quality flag are available. They indicate the results of the QA/QC procedures applied to sensor data. The data quality flags are provided for users to be able to filter sensor data in order to ensure using only robust data, or in order to test the output of their own filtering procedures compared to the one provided with the data. The name of the columns with the data flag has a format of Sensor_Flag where Sensor includes: CO_A4_P1, D300, NO_B4_P1, NO2_B43F_P1, OX_A431_P1, 5301CAT, 5301CST, 5325CAT, 5325CST, 5310CAT, 5310CST, OPCN3PM1, OPCN3PM25 or OPCN3PM10. The data flags can contain the following labels:

- empty labels: indicates valid raw data after all QA/QC procedures are applied;
- “W” indicates data flagged for warming up of AirSensEUR sensor system after a cold start, any reboot of the AirSensEUR sensor systems or restart of AirSensEUR data acquisition. Warming up time is required for allowing the sensor to reach the full sensor response capacity. Table 11 gives the suggested warming time for all sensor in the row “Warming”;

**Table 11:** Parameters of the data filtering procedure.

	CO_A4	D300	NO_B4	NO2_B43F	OX_A431	PMS 5003*	OPC-N3*	BMP 280	SHT31HE	SHT31TE	SHT31HI	SHT31TI
Warming, hr	4	4	30	4	4	4	4	1	1	1	1	1
T.min, °C	−20	−20	−20	−20	−20	−20	−20	−40	−40	−40	−40	−40
T.max, °C	40^+^	40^+^	40^+^	40^+^	40^+^	40^+^	40^+^	65	65	65	65	65
rh.min, %	15	15	15	15	15	10	10	0	0	0	0	0
rh.max, %	100	100	100	100	100	100	100	100	100	100	100	100
Window, min	181	181	181	181	181	181	181	181	181	181	181	181
Threshold	75	8	75	20	20	20	20	20	20	20	20	20
Min_values	−386, nA	100, ppm	−501, nA	−3664, nA	−3664, nA	0, µg.m^−^³	0, µg.m^−^³	800, hPa	0, %	−20, °C	0, %	−20, °C
Max_values	2263, nA	3000 ppm	1441, nA	193, nA	193, nA	300, µg.m^−^³	300, µg.m^−^³	1060, hPa	100, %	65, °C	100, %	65, °C

*PM_10_, PM_2.5_ and PM_1_.

^+^45 for Zagreb.

- “T.min” or “T.max” and/or “Rh.min” or “Rh.max” indicates data outside temperature and/or relative humidity limits. These four thresholds were empirically determined, either from experience or laboratory experiments^4,17,18^. Extreme temperature and humidity may affect sensor performance resulting in inaccurate, noisy and/or questionable data. The upper and lower bounds of temperature and relative humidity were set to filter sensor data out, as sensor may behave incorrectly outside these bounds, e. g. OPC-N3 overestimating PM mass concentration for high relative humidity. The suggested limits of acceptability of temperature (T.min and T.max) and relative humidity (rh.min and rh.max) are given in Table 11.

- “Low_values” or “High_values”: indicates data flagged when data were lower than the minimum acceptable values (Min_values in Table 11) or higher than maximum acceptable values (Max_values in Table 11. Both “Low_values” and “High_values” corresponded to the limits due to the range of the AirSensEUR data acquisition, the operational range of the sensors or impossibilities of air pollution levels.
- “OutliersMin” or “OutliersMax”: indicates data flagged when applying the outlier filtering procedure. Occasional outliers in sensor data, might happen due to several reasons. The detection of outliers at all x_i_ in dataset was performed using an Hampel filter based on the Mean Absolute Deviation MAD_i_, computed using Eq. 1 over a rolling time windows centred on x_i_ including all x_j_ values within a time Window (see Window in Table 11). Subsequently, MAD_i_ was expanded with the Threshold factor (see Threshold in Table 11) in order to determine limits of acceptance for x_i_. The Threshold factors were set to 20 for all sensors except for CO_2_ (8), CO and NO (75 each), for which the concentration levels can change rapidly). For any sensor data x_i_ lower than the lower limit defined in Eq. 2, OutliersMin was added to the Sensor_flag column. For any sensor data x_i_ exceeding the higher limit defined in Eq. 3, OutliersMax was added to the Sensor_flag column. A critical point of outlier detection using MAD is to determine the time window such that spikes in data shall be recognized to be real or outlier in measurements. The time window was set to 3 hrs (181 data points).

1 $${{\rm{MAD}}}_{{\rm{i}}}={\rm{median}}\left|{{\rm{x}}}_{{\rm{j}}}-{\rm{median}}\left({{\rm{x}}}_{{\rm{j}}}\right)\right|{\rm{in}}\;{\rm{a}}\;{\rm{rolling}}\;{\rm{window}}\;{\rm{of}}\;{{\rm{x}}}_{{\rm{j}}}\;{\rm{data}}$$

2 $${\rm{OutliersMin}}:{{\rm{x}}}_{{\rm{i}}} < {\rm{median}}\left({{\rm{x}}}_{{\rm{j}}}\right)-{\rm{threshold}}\;{{\rm{MAD}}}_{{\rm{i}}}$$

3 $${\rm{OutliersMax}}:{{\rm{x}}}_{{\rm{i}}} > {\rm{median}}\left({{\rm{x}}}_{{\rm{j}}}\right)+{\rm{threshold}}\;{{\rm{MAD}}}_{{\rm{i}}}$$

- “Inv” indicates sensor data flagged as invalid. A few invalid sensors were manually flagged as they corresponded to move of the sensor systems, unknown location of sampling, periods of maintenance or calibration of the reference analysers and a few malfunctions of sensors, e.g., insects in OPC, aging of chemical sensor, general failure of sensors. “Inv” is sometimes added to the flag of sensor data although sensor data are correct while because of maintenance or calibration of reference analysers, comparison of reference and sensor data should not be carried out.

For sensor data that do not satisfy two or more of the criteria listed above, the Sensor_flag consist of the concatenation of the flag labels, with a comma separation between quotes.

For the OPC-N3 sensors, the rh.max was initially set to 70%, as suggested by the manufacturer. However, based on testing it was later set to 100% in order to keep all data in case they might be used later on for calibration with Kohler models^18–20^. The Kohler model requires higher relative humidity than 70% for achieving the best possible fit. Several tests were performed to determine the rh.max for multi linear and Kohler fittings. The results showed that for multi-linear and Kohler fittings, the best predictions were obtained by rh.max of 70% and 100%, respectively.

All values of parameters for filtering are given in Table 11. They are mainly derived from experience (i. e. Warming, Window and Threshold). One may notice that the values of T.min, T.max, rh.min and rh.max do not discard many outliers. However, these parameters could be set to more stringent values that could be useful for filtering for example high relative humidity for PM sensors or high temperature that affect NO_B4 sensors. The Min_values and Max_values for sensors CO_A4, NO_B4, NO2_B43F and OX_A431 are constrained by the electronics of the AirSensEUR data acquisition. They should not be changed. Conversely, the Min_values and Max_values for sensors D300, PMS5003, OPC-N3, BMP280, SHT31HE, SHT31HI, SHT31TE and SHT31TI are set using expected reading and can be fine-tuned in order to discard outliers. Finally, the majority of values given in Table 11 are not absolute rules and data users can experiment with new values in order to improve the filtering procedure.

## Usage Notes

For users who would like to study minute-level sensor data against minute-level reference data, some lag between sensor and reference time series can be an obstacle. Although AirSensEUR time series refers to Coordinated Universal Time drawn from GPS or GPRS or WIFI, this does not exclude different response time of LCS and reference analyser and other mistakes in reporting time of data series. Before any data treatment, it is strongly suggested to apply a lag correction for the sensor and reference data series being studied. Lag between two data series can be estimated using the output of cross correlation function (CCF)^21^. The maximum CCF can be estimated using the Find_Max_CCF function in “151016 Sensor_toolBox.R” file (https://github.com/ec-jrc/airsenseur-calibration).

## Data Availability

Code for filtering data is available in the file Functions4ASE.R at https://github.com/ec-jrc/airsenseur-calibration. The whole QC/QC filtering is carried out using the function Filter_Sensor_Data() in the Function4ASE.R file. Filter_Sensor_Data() includes the flagging of data during warming using function Warm_Index(), the flagging of data outside temperature and relative humidity using function TRh_Index, the flagging of outliers using function Outliers_Sens() and the flagging of invalid data using function Inv_Index(). Code for calibrating AirSensEUR sensor box is available in file CompareModels.R at https://github.com/ec-jrc/airsenseur-calibration/tree/master/Auto_Calibration. Explanation is given to use this script in the AirSensEUR Guidance report^7^.
